# Association of adverse childhood experiences and cortical neurite density alterations with posttraumatic stress disorder symptoms in autism spectrum disorder

**DOI:** 10.3389/fpsyt.2023.1215429

**Published:** 2023-09-08

**Authors:** Soichiro Kitamura, Kiwamu Matsuoka, Masato Takahashi, Hiroaki Yoshikawa, Akihiro Minami, Hiroki Ohnishi, Rio Ishida, Toshiteru Miyasaka, Yumi Tai, Tomoko Ochi, Toshihiro Tanaka, Manabu Makinodan

**Affiliations:** ^1^Department of Psychiatry, Nara Medical University School of Medicine, Kashihara, Japan; ^2^Department of Functional Brain Imaging Research, National Institute Radiological Sciences, National Institutes for Quantum and Radiological Science and Technology, Chiba, Japan; ^3^Department of Radiology and Nuclear Medicine, Nara Medical University, Kashihara, Japan

**Keywords:** autism spectrum disorder, posttraumatic stress disorder, NODDI, adverse childhood experience, neurite density

## Abstract

**Background:**

Posttraumatic stress disorder (PTSD) can be a source of significant social and daily distress in autism spectrum disorder (ASD). Compared to typically developed (TD) individuals, people with ASD are at an increased risk of adverse childhood experiences (ACEs), which can result in abnormal neuronal development. However, whether or how ACEs influence abnormal neural development and PTSD symptoms in ASD has not been fully elucidated.

**Methods:**

Thirty-nine TD individuals and 41 individuals with ASD underwent T1-weighted magnetic resonance imaging and neurite orientation dispersion and density imaging (NODDI), with axonal and dendritic densities assessed in terms of the orientation dispersion index and neurite density index (NDI), respectively. Voxel-based analyses were performed to explore the brain regions associated with PTSD symptoms, and the relationships between the severity of ACEs and PTSD symptoms and NODDI parameters in the extracted brain regions were examined.

**Results:**

There was a significant positive association between PTSD symptom severity and NDI in the bilateral supplementary motor area; right superior frontal, left supramarginal, and right superior temporal gyrus; and right precuneus in the ASD group, but not in the TD group. ACE severity was significantly associated with NDI in the right superior frontal and left supramarginal gyrus and right precuneus in the ASD group. Moreover, NDI in the right precuneus mainly predicted the severity of PTSD symptoms in the ASD group, but not the TD group.

**Conclusion:**

These results suggest that ACE-associated higher neurite density is of clinical importance in the pathophysiology of PTSD symptoms in ASD.

## 1. Introduction

Autism spectrum disorder (ASD) is a common neurodevelopmental disorder characterized by poor social communication skills, difficulties related to flexible thinking and behavior, and hyper- and hypo-sensitivity toward external sensory stimuli (1). These characteristics often make it difficult to develop personal relationships and act flexibly in daily situations related to academic and social life; moreover, this inability to socially adapt appropriately can cause increased stress and loss of confidence in affected individuals, often subsequently evoking other mental health problems (2–4). In fact, it is well known that mental health-related comorbidities, such as depression, anxiety disorders, and substance use disorders, often afflict individuals with ASD (5–7).

Posttraumatic stress disorder (PTSD) is characterized by repetitive and unconquerable intrusive mental imagery; hyperarousal, including overstressing; emotional instability; and difficulty in concentrating as a result of experiencing severely traumatizing life events (1, 7). Therefore, patients with PTSD tend to avoid stressful stimuli that they correlate with these traumatic events. Individuals with ASD who experience violence, bullying, and scolding often show intrusive re-experiencing, overstressing, poor thinking, and concentration when faced with stressful life events, similar to typical PTSD patients (8–10). However, although such PTSD symptoms often contribute to poor social functioning in individuals with ASD, there are no systematic and effective treatments available.

Exposure to maltreatment, such as abuse and neglect from parents and/or caregivers and bullying at school during childhood and adolescence, has been shown to be involved in social and psychological dysfunction (11, 12). These adverse childhood experiences (ACEs) are considered as risk-factors for the incidence of mental illnesses such as depression, anxiety disorder, and PTSD in adulthood (13–15). It is possible that exposure to ACEs affects normal neurodevelopment during childhood and adolescence, consequently forming the pathological basis for mental illnesses in adulthood. This has also been demonstrated by neuroimaging studies that examined the association between exposure to ACEs and brain structure and function, as their findings indicate that ACE-related neural network abnormalities can be the pathological basis of mental illnesses, including PTSD, in adulthood (16–19). However, gray matter microstructural abnormalities associated with PTSD symptoms in ASD have not yet been fully elucidated. Regarding PTSD symptoms in individuals with ASD, we recently reported the association between intrusive re-experiencing and reduced gray matter volume in the precuneus (20). Neuroimaging studies have also shown that the structural abnormalities in PTSD involve reduced subcortical gray matter volume and cortical thickness (21–24). Recently, Berman et al. used the diffusion tensor imaging approach to demonstrate the presence of cortical and subcortical gray matter microstructural abnormalities in PTSD patients (25).

Neurite orientation dispersion and density imaging (NODDI) is an effective neuroimaging technique that has proven to be useful for intracellular, extracellular, and cerebrospinal fluid biophysical modeling. The derived models enable the *in vivo* quantification of the angular variation of neurites and axonal and dendritic densities in terms of the orientation dispersion index (ODI) and neurite density index (NDI), respectively (26, 27). NODDI can be used to detect microstructural alterations in neuronal morphology, specifically in the cortical and subcortical gray matter. Recently, several research groups, including ours, have examined the gray matter microstructural changes related to psychiatric disorders (28–32). Thus, NODDI could facilitate the identification of the characteristic neuronal morphology alterations associated with psychiatric symptoms in ASD.

Previously, we have examined the abnormal neurite density associated with sensory over-responsivity and ACEs (33), which suggested the clinical importance of gray matter microstructural abnormality in ASD. Exploring the pathological basis of PTSD symptoms is of clinical importance for accurately assessing their impact in ASD. Moreover, given that individuals with ASD are at increased risk of exposure to ACEs, identifying whether and how ACEs influence PTSD symptoms and gray matter microstructure could also promote considerations of early intervention to prevent maltreatment of children with ASD. The objective of this study was to investigate gray matter microstructural alterations associated with PTSD symptoms across the domains of intrusive thinking, hyperarousal, and avoidance symptoms in ASD. We also explored whether the level of stress in early life, including due to sexual, physical, and emotional abuse and neglect, is associated with gray matter microstructural alterations in various brain regions and PTSD symptoms in ASD. Elucidating the brain regions with pathological changes that are associated with PTSD symptoms could help in clarifying the pathophysiology of PTSD symptoms and in developing appropriate clinical interventions in ASD.

## 2. Methods

### 2.1. Participants

We enrolled adult participants of 39 typically developed (TD) individuals (Age; 27.5 ± 5.4 years) and 41 individuals with ASD (Age; 28.2 ± 6.5 years) in this study. The current samples have been used in our previous study (33). Individuals with ASD were recruited at the outpatient clinic of the Department of Psychiatry, Nara Medical University Hospital and the affiliated psychiatric clinic. They were diagnosed by two trained psychiatrists based on the Diagnostic and Statistical Manual of Mental Disorders, Fifth Edition criteria and the Japanese version of the Autism Diagnostic Observation Schedule-Second Edition (ADOS-2) (34). They were also evaluated using the Japanese version of the Autism Spectrum Quotient (AQ-J) test, and only those with a score ≥ 33 were enrolled (35, 36). Among the individuals with ASD, 15 subjects had other neuropsychiatric comorbidities, although none were diagnosed with PTSD. Twenty-two individuals with ASD were receiving psychotropic medications during the study period.

The TD participants included students and hospital and university staff of Nara Medical University with no history of psychiatric, neurological, or developmental disorders as assessed based on the Mini-International Neuropsychiatric Interview. We also estimated the full-scale intelligence quotient (IQ) of each participant using the similarities and symbol search subsets of the Wechsler Adult Intelligence Scale-Third Edition (37). The socioeconomic status was evaluated by the Brief rating scale of socioeconomic status for both individuals with ASD and TD individuals (38). The exclusion criteria for both the ASD and TD groups were a total IQ < 70 and structural brain abnormalities on T1-weighted magnetic resonance (MR) images.

### 2.2. Measures

#### 2.2.1. Assessment of PTSD symptoms

The severity of PTSD symptoms was measured using the Japanese version of the Impact of Event Scale-Revised (IES-R-J) (39). The IES-R-J is commonly used to evaluate self-reported posttraumatic stress symptoms, such as intrusion (8 items), hyperarousal (6 items), and avoidance (8 items), after a traumatic experience. Participants rated each questionnaire item with respect to one past life event which force you feeling great discomfort on a scale of 0 to 4 (0 = not at all, 4 = extremely); the IES-R-J total and subscale (intrusion, hyperarousal, and avoidance) scores were calculated accordingly, with higher scores reflecting greater severity. Since the participants may feel unwell during the examination, medical staff were ready to follow up the participants’ condition carefully.

#### 2.2.2. Evaluation of ACEs

The severity of ACEs was evaluated using the Japanese version of the Child Abuse Trauma Scale (CATS) (40, 41). The CATS questionnaire consists of 38 self-rated items and retrospectively assesses the frequency of adverse event experiences in childhood and adolescence in five categories: sexual abuse, physical abuse, emotional abuse, neglect, and others. Participants rated each questionnaire item on a scale of 0–4 (0 = never; 4 = always). The total CATS score can range from 0 to 152, with a higher score reflecting greater severity. A previous study has examined that the CATS showed reliable internal consistency (Cronbach’s *α* = 0.63–0.90) and test–retest reliability (*r* = 0.71–0.91) (40).

These psychological tests were individually administrated with careful explanations by the evaluators considering the possibility that individuals with ASD experience difficulties to complete self-questionnaires as previously reported (42).

This study was prospectively reviewed and approved by the institutional review board of Nara Medical University and performed in accordance with the relevant guidelines and regulations. Written informed consent was obtained from all participants before enrollment in the study.

#### 2.2.3. Magnetic resonance imaging

A Siemens MAGNETOM Verio scanner (Siemens Healthcare, Erlangen, Germany) was used to acquire 3 T MR images. Participants were scanned using a 3D T1-weighted gradient echo sequence [repetition time (TR) = 1900 ms; echo time (TE) = 2.54 ms; field of view (FOV) = 256 × 256 mm; acquisition matrix = 256 × 256; 208 contiguous 1.0-mm thickness axial slices]. A two-shell diffusion MR imaging (MRI) protocol was employed to obtain NODDI data. Diffusion MRI scans were acquired using an echo-planar imaging sequence (TR = 17,500 ms; TE = 93 ms; *b* value = 0, 1,000, and 2000 s/mm^2^; acquisition matrix = 114 × 114; 75 contiguous 2.0-mm thickness axial slices; no intersection gap; flip angle = 90°). The reconstruction matrix was the same as the acquisition matrix, and 2 × 2 × 2 mm isotropic voxel data were obtained. A motion-probing gradient was applied in 30 directions for two *b* values: 1000 and 2000 s/mm^2^. The phase-encoding direction for these datasets was anterior to posterior. All participants were observed to ensure that they did not move during MRI. To ensure that the acquired MR images were of appropriate quality, individual scans were visually examined before any image processing step was performed.

#### 2.2.4. Diffusion tensor imaging

Diffusion-weighted images were processed using the functional MRI of the brain (FMRIB) software library (FSL) version 6.0.0 (FMRIB Center, Department of Clinical Neurology, University of Oxford, Oxford, England)^1^. Brain tissue data was extracted using the FSL brain extraction tool. Diffusion-weighted images for each of the 30 directions were corrected for eddy currents and head motion (43). To assess head motion, we evaluated the root mean square (RMS) deviation of absolute intervolume displacement with respect to *b* = 0 images from intraparticipant registration parameters using the FSL rmsdiff tool (44). The average displacement distance between each consecutive pair of 61 volumes was calculated for each participant. The NODDI model was fitted using the NODDI toolbox^2^ running in MATLAB (MathWorks, Natick, MA, United States), and the ODI and NDI value maps were derived.

Each ODI and NDI image was co-registered with the corresponding T1-weighted images, which were segmented into gray and white matter and cerebrospinal fluid. Subsequently, each gray matter image was spatially normalized to the Montreal Neurological Institute (MNI) 152 standard space using a Diffeomorphic Anatomical Registration Through Exponentiated Li Algebra algorithm (45). Moreover, based on the transformation matrix, individual ODI and NDI images were also spatially normalized to the MNI 152 standard space and smoothed using an 8-mm full-width at half-maximum isotropic Gaussian kernel. Voxel-based analyses were performed to investigate the brain regions associated with each IES-R-J subscale score in both the TD and ASD groups. ODI and NDI image processing and voxel-based analyses were conducted using the Statistical Parametric Mapping software (SPM 12, Wellcome Trust Centre for Neuroimaging, London, United Kingdom).

### 2.3. Statistical analysis

Between group differences in demographic characteristics (age, IQ, duration of education) and AQ-J, ADOS-2, IES-R-J total and subscale and CATS total scores were examined using the Mann–Whitney U test for nonparametric data (*p <* 0.05). Fisher’s exact test was employed to examine differences in sex and handedness between the two groups (*p <* 0.05).

For voxel-based analysis, we applied multiple linear regression to identify the brain regions associated with each IES-R-J subscale score in the ASD group, with duration of education and use of psychotropic medication as covariates. Similarly, in the TD group, duration of education was used as the covariate for the voxel-based whole-brain regression analysis. A liberal threshold of *p <* 0.001 was used to obtain clusters with a family-wise error of *p <* 0.01.

Correlation analysis was used to explore the relationships between the CATS total score and each IES-R-J subscale score in both the ASD and TD groups (Pearson correlation analysis for parametric data and Spearman rank correlation for nonparametric data). Statistical significance was set at *p <* 0.017 using Bonferroni correction for multiple comparison. Moreover, partial correlation analyses were performed to examine the relationships between the CATS total score and NODDI values in the extracted brain regions in the voxel-based analysis; the covariates were duration of education and use of psychotropic medication for the ASD group and duration of education for the TD group. Statistical significance was set at *p <* 0.05/n (where “*n*” is the number of extracted brain regions in the voxel-based analyses) using Bonferroni correction for multiple comparison.

Additionally, to determine whether the severity of ACEs and/or gray matter microstructural alterations in the extracted brain regions were associated with that of PTSD symptoms, stepwise multiple regression analyses was performed with each IES-R-J subscale score as the dependent variable and CATS total score and NODDI values in the extracted brain regions as independent variables in the ASD and TD groups, respectively; statistical significance was set at *p <* 0.05.

The normality of each dataset was evaluated using the Shapiro–Wilk test. Statistical analyses for the group comparison of demographic characteristics, regional NDI values, and gray matter volume; correlation analyses; and multiple regression analyses were performed using SPSS version 27 (IBM Inc., Armonk, NY, United States).

## 3. Results

### 3.1. Demographic data

**Figure 1:**
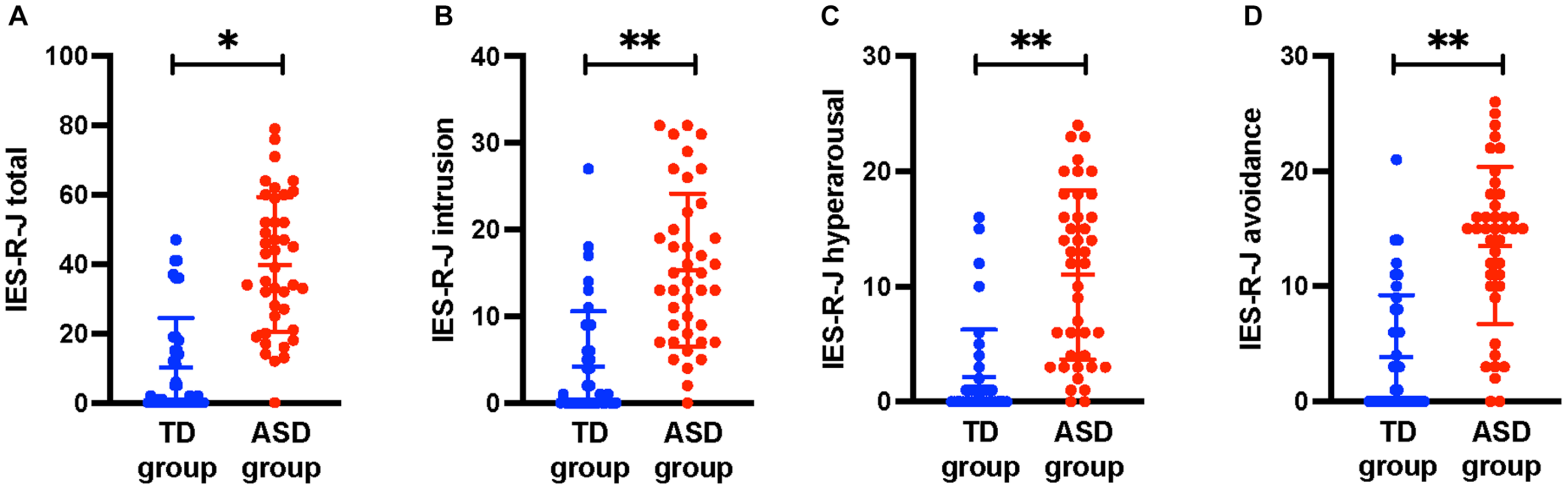
Comparisons of IES-R-J total and subscale scores between the ASD and TD groups. The IES-R-J **(A)** total and **(B–D)** subscale scores were significantly higher in the ASD group than in the TD group. *, *p* < 0.05; **, *p* < 0.017. IES-R-J, the Japanese version of the impact of event scale-revised; ASD, autism spectrum disorder; TD, typically developed.

Table 1 shows a comparison of the demographic characteristics between the ASD and TD groups. The duration of education was significantly shorter and AQ-J score was significantly higher in the ASD group than in the TD group (*p* < 0.05). There were no significant differences in age, sex, handedness, IQ, and socioeconomic status between the two groups (*p* < 0.05). Regarding the severity of ACEs, the ASD group had significantly higher CATS total (*p* < 0.05) score than the TD group. Figure 1 shows the comparison of IES-R-J total and subscale scores between the ASD and TD groups. The total score was significantly higher in the ASD group (ASD, 39.9 ± 19.5; TD, 10.2 ± 14.2; U = 1,417; *p* < 0.001); the intrusion (ASD, 15.3 ± 8.8; TD, 4.2 ± 6.4; U = 1,383; *p* < 0.001), hyperarousal (ASD, 11.0 ± 7.3; TD, 2.1 ± 4.1; U = 1,405; *p* < 0.001), and avoidance (ASD, 13.5 ± 6.8; TD, 3.9 ± 5.4; U = 1389.5; *p* < 0.001) subscale scores were also significantly higher than those in the TD group. There was no significant difference between the groups in terms of head motion based on the average RMS distance (ASD, 4.6 ± 1.4 mm; TD, 4.2 ± 0.7 mm; U = 890.5; *p =* 0.38).

**Table 1:** Demographic characteristics of the study participants.

	ASD group (*n* = 41)	TD group (*n* = 39)	*T* or *U* or *c*^2^	*p*
Age, mean (SD)	28.2 (6.5)	27.5 (5.4)	829	0.78
Years of schooling, mean (SD)	14.8 (2.7)	16.2 (2.1)	571	0.023^*^
Sex, male (%)	30 (73.2)	28 (71.8)	0.019	0.89
Handedness, right (%)	38 (92.7)	39 (100)	3	0.085
IQ, mean (SD)	100.2 (13.7)	104.5 (9.8)	646	0.14
AQ-J score, mean (SD)	36.3 (3.2)	19.3 (6.8)	1,599	< 0.001^*^
ADOS-2 score, mean (SD)	15.8 (3.2)	N/A	N/A	N/A
CATS score, mean (SD)	49.1 (28.4)	22.9 (19.7)	1325.5	< 0.001^*^

TD, typically developed; ASD, autism spectrum disorder; IQ, intelligence quotient; AQ-J, Japanese version of the autism spectrum quotient test; ADOS-2, autism diagnostic observation schedule-second edition; IES-R-J, Japanese version of the impact of event scale-revised; CATS, child abuse and trauma scale; SD, standard deviation. ^*^, *p* < 0.05.

Table 2 shows the correlations between individual IES-R-J subscale scores and CATS total score. There were significant positive correlations between the CATS total score and IES-R-J intrusion and hyperarousal subscale scores (*p* < 0.017), but not avoidance subscale score (*p* > 0.017) in the ASD group. On the other hand, there were significant positive correlations between the CATS total score and IES-R-J intrusion, hyperarousal, and avoidance subscale scores in the TD group (*p* < 0.017).

**Table 2:** Associations between severity of total CATS and each IES-R-J subscale score.

	CATS	
	ASD	TD
IES-R-J		
intrusion	0.51 (0.001)^*^	0.47 (0.003)^*^
hyperarousal	0.40 (0.013)^*^	0.47 (0.003)^*^
avoidance	0.12 (0.47)	0.52 (0.001)^*^

ASD, autism spectrum disorder; TD, typically developed; IES-R-J, Japanese-language version of the impact of event scale-revised; CATS, child abuse and trauma scale. Each value shows r or r_s_ (*p* value). ^*^: *p* < 0.017.

### 3.2. Identifying brain regions with significant association between NODDI values and IES-R-J scores in the ASD group

**Figure 2:**
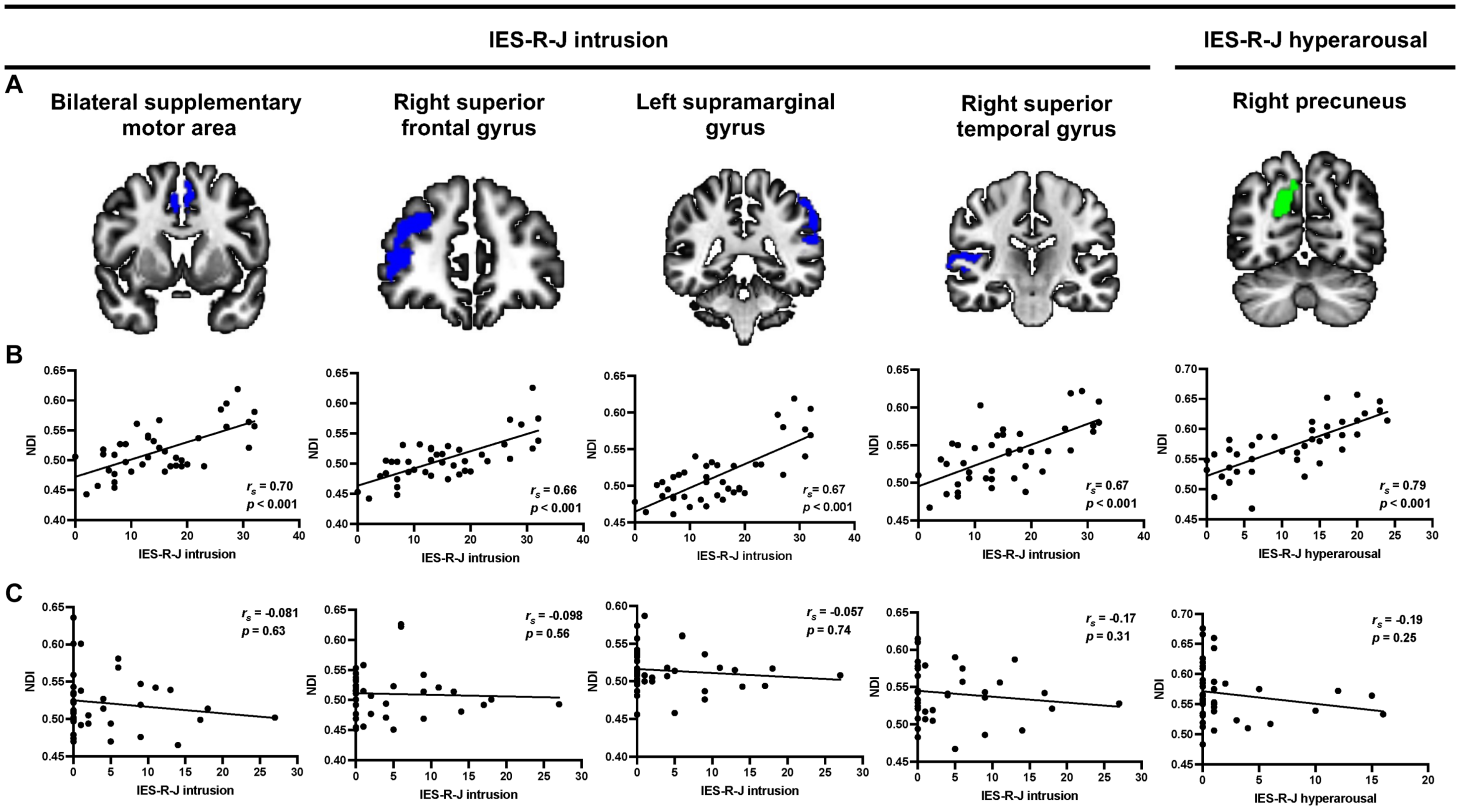
Representative voxel-based multiple regression analysis between each IES-R-J subscale score and NDI values. **(A,B)** Severe IES-R-J intrusion subscale score was significantly associated with increased NDI values in the bilateral supplementary motor area, right superior frontal gyrus, supramarginal gyrus, and right superior temporal gyrus (blue) in the ASD group. Additionally, there was a significant positive association between severe IES-R-J hyperarousal subscale score and NDI values in the right precuneus (green) in the ASD group. **(C)** There were no significant associations between IES-R-J subscale scores and NDI values in the abovementioned brain regions in the TD group. IES-R-J, the Japanese version of the impact of event scale-revised; NDI, neurite density index; ASD, autism spectrum disorder; TD, typically developed.

Figure 2 shows the results of the voxel-based whole-brain analyses of the associations between IES-R-J subscale scores and NODDI values. There were significant positive correlations between the IES-R-J intrusion subscale score and NDI values in the bilateral supplementary motor area [(*x*, *y*, *z*) = (−6, 4, 50), cluster voxel size = 473, *T* = 4.8, *p* < 0.001], right superior frontal gyrus [(*x*, *y*, *z*) = (30, 50, 10), cluster voxel size = 1,196, *T* = 6.4, *p* < 0.001], left supramarginal gyrus [(x, y, z) = (−58, −40, 36), cluster voxel size = 2056, *T* = 5.9, *p* < 0.001], and right superior temporal gyrus [(*x*, *y*, *z*) = (66, −28, 8), cluster voxel size = 497, *T* = 5.1, *p* < 0.001] in the ASD group. Moreover, the IES-R-J hyperarousal subscale score was significantly and positively correlated with NDI values in the right precuneus [(*x*, *y*, *z*) = (18, −66, 28), cluster voxel size = 480, *T* = 5.0, *p* < 0.001] in the ASD group. After controlling for the effect of age in addition to duration of education and use of psychotropic medication, the association between the IES-R-J hyperarousal subscale score and NDI values in the right precuneus remained still significant; but no significant brain regions were associated with the IES-R-J intrusion subscale score. Then, in case of controlling for the effect of sex and intracranial volume in addition to duration of education and use of psychotropic medication, the brain regions associated with IES-R-J intrusion subscale (right superior frontal and left supramarginal gyri) and IES-R-J hyperarousal (right precuneus) remained still significant. There were no significant associations between the IES-R-J avoidance subscale score and NDI values or between any IES-R-J subscale score and ODI values in the ASD group. Voxel-based analyses showed that there was no significant association between any IES-R-J subscale score and NODDI values in the abovementioned brain regions in the TD group. Additionally, group comparisons showed no significant NDI values in these brain regions in either the ASD or TD group (Supplementary Table S1).

### 3.3. Correlations between NDI values in the brain regions associated with IES-R-J and CATS scores

**Figure 3:**
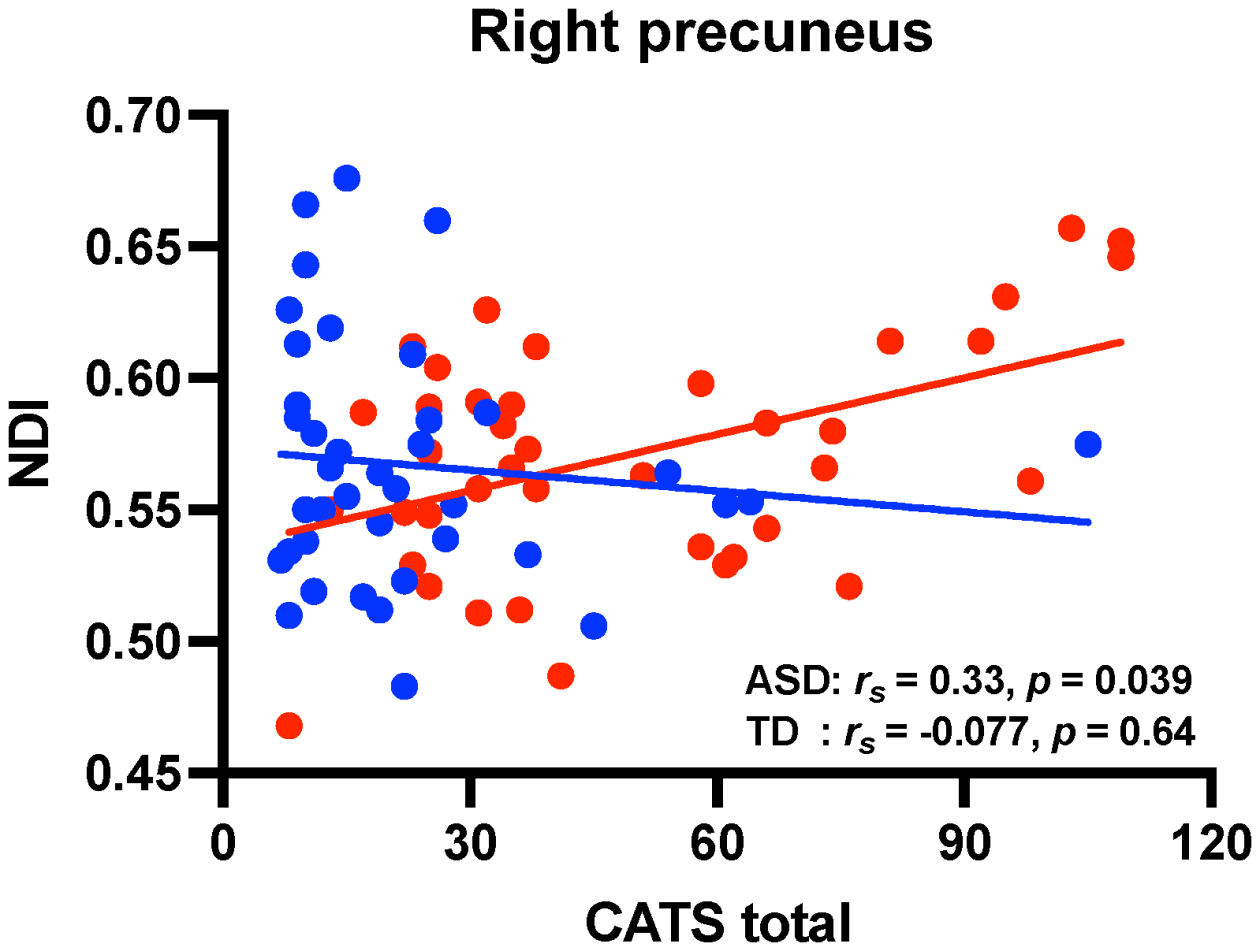
Correlation analyses between CATS score and NDI values in the brain regions associated with PTSD symptoms. Scatter plots showing the relationships between CATS total score and NDI values (red circles, individuals with ASD; blue circles, TD individuals). There were significant positive correlations between CATS total score and NDI values in the right precuneus in the ASD group. On the contrary, there were no significant correlations in the TD group. CATS, child abuse trauma scale; NDI, neurite density index; PTSD, posttraumatic stress disorder; ASD, autism spectrum disorder; TD, typically developed.

Figure 3 shows scatter plots depicting the relationships between NDI values in the extracted brain regions mentioned above and CATS total score in both groups. Four brain regions associate with IES-R-J intrusion subscale scores were extracted in the voxel-based whole-brain analysis [statistical significance was set at *p* < 0.0125 (0.05/4)]. The CATS total score was positively correlated with NDI values in the right precuneus (IES-R-J hyperarousal) in the ASD group. There were no significant correlations between the CATS total score and NDI values in the brain regions associated with IES-R-J intrusion (Supplementary Table S2). Unlike in the ASD group, there was no significant correlations between CATS total score and NDI values in any of the brain regions (Supplementary Table 2) in the TD group.

### 3.4. Cats scores and NDI values in the IES-R-J associated brain regions predicted IES-R-J scores in ASD

Using multiple regression analyses, we explored whether any CATS total score or NDI values in the extracted brain regions could predict the severity of IES-R-J subscale score in the ASD group (Table 3). Regarding the IES-R-J hyperarousal subscale score in the ASD group, we defined NDI value in the right precuneus and CATS total score as independent variables in terms of predicting IES-R-J hyperarousal subscale score severity. NDI value in the right precuneus significantly predicted severity of the IES-R-J hyperarousal subscale score (*p* < 0.05). Then, since the socioeconomic status and existence of psychiatric comorbidity can influence each PTSD symptom, we added these factors to independent valuables for the multilinear regression analysis. As a result, the socioeconomic status and existence of psychiatric comorbidity did not predict the severity of the IES-R-J hyperarousal subscale score (*p* < 0.05) (Supplementary Table S3).

**Table 3:** Multiple regression analysis: each IES-R-J hyperarousal subscale vs. CATS total score and regional NDI in ASD.

Predictor	*R* ^2^	Δ*R*^2^	*F*	*B*	*b*	*p*
IES-R-J hyperarousal	0.19	0.17	9.0^*^			
CATS total score				0.096	0.098	0.084
NDI in the right precuneus				67.8	0.43	0.005^*^

IES-R-J, Japanese-language version of the impact of event scale-revised; CATS, child abuse trauma scale; NDI, neurite density index; ASD, autism spectrum disorder; ACE, adverse childhood experiment; R^2^, coefficient of determination; ΔR^2^, adjusted coefficient of determination; B, partial regression coefficient; SE B, standard error of B; b, standard regression coefficient. ^*^: *p* < 0.05.

## 4. Discussion

To the best of our knowledge, this is the first study to explore PTSD-associated gray matter microstructural alterations in individuals with ASD. Our results showed that individuals with ASD demonstrated significant associations between severity of PTSD symptoms and higher NDI values in the bilateral supplementary motor area; right superior frontal, left supramarginal, and right superior temporal gyrus; and right precuneus, which were not observed in TD individuals. We hypothesize that higher neurite density in these brain regions is involved in the manifestation of PTSD symptoms in individuals with ASD. Moreover, although there were significant correlations between severity of PTSD symptoms and CATS scores in both groups, significant relationships between NDI values in the PTSD-associated brain regions and CATS scores were observed only in the ASD group. These findings suggest that exposure to severe ACEs may be closely involved in the higher cortical neurite density in individuals with ASD compared to that in TD individuals.

Individuals with ASD showed significant associations between severity of intrusion and higher neurite density across the frontal, parietal, and temporal brain regions. The supplementary motor area is associated with both motor control and proper cognitive control and to contribute to the neural network mediating cognitive control (46). The frontal and supramarginal gyrus have been implicated in memory encoding and retrieval and precise recognition of past experiences (47–49). The superior temporal gyrus has been associated with audiovisual integration of emotions, and is connected to the amygdala, which is known to be clinically important in PTSD pathophysiology (50, 51). These frontal, temporal, and parietal brain regions are functionally linked and involved in autobiographic memory retrieval processing, and abnormalities in this process have been shown to be associated with PTSD (47, 52, 53). Therefore, abnormal processing of autobiographical memory retrieval and emotion could be associated with intrusion in ASD. Additionally, individuals with ASD showed an association between severity of hyperarousal and higher neurite density in the precuneus. The precuneus is involved in episodic memory retrieval, self-consciousness, and self-related mental representations (54, 55). Thus, reduced precuneus functioning may be associated with abnormal processing of self-consciousness and mental representation, and consequently with irritability, sleep disruption, and difficulty in concentrating in ASD. On the other hand, no significant association was found between avoidance and NDI in individuals with ASD. This may be because avoidance is a coping behavior influenced mainly by intrusion and hyperarousal (56, 57), and to a lesser extent by cortical microstructure changes. Thus, despite the significant association between higher neurite density and PTSD symptom severity in individuals with ASD, there were no significant differences in NDI values in the PTSD-associated brain regions between the groups, which was an unexpected finding. Based on this result, it can be supposed that, compared to TD individuals, individuals with ASD have potential vulnerabilities in the PTSD-associated brain regions. Since NDI values in the PTSD-associated brain regions were significantly correlated with CATS score, cortical neurite density can be susceptible to severity of ACEs in individuals with ASD (58). Our ASD samples in this study showed relatively broad range of CATS scores compared to TD individuals, which may have led to wide variability in NDI value in the PTSD-associated brain regions. This could be the reason why NDI values were not different between individuals with ASD and TD individuals.

Our findings showed that exposure to severe ACEs was associated with severity of PTSD symptoms in individuals with ASD. Previous studies have reported that exposure to early life trauma associated with PTSD in adults with ASD (10, 59, 60), which was consistent with our findings. Additionally, TD individuals showed significant associations between severity of ACEs and that of each PTSD symptom. There have been previous reports on the association between early life stress and mental health in healthy adults (61–64), which was consistent to our findings. Although the etiological basis of ACE-induced psychological symptoms after adulthood remains unclear, the involvement of hypothalamic–pituitary–adrenal axis dysregulation (65–67) and/or systemic inflammation (68–70) has been hypothesized. However, regression analyses showed that severity of PTSD symptoms, rather than that of ACEs, was critically predicted by higher neurite density in PTSD-associated brain regions in the ASD group, but not the TD group. This suggests that the etiological basis of PTSD symptoms differs between the groups, and that gray matter microstructural alteration is of clinical importance for the manifestation of PTSD symptoms in individuals with ASD. Accordingly, treatment strategies involving functional modification of PTSD-associated brain regions, such as neuromodulation, could prove to be effective clinical interventions for ASD.

Severity of CATS scores showed significant associations with higher NDI values in the PTSD-associated brain regions in individuals with ASD. Exposure to severe childhood maltreatment has been reported to be involved in abnormalities of gray matter development (71–73), and a previous study has examined gray matter density associated with child abuse (74), which were in line with our findings. Exposure to ACEs is suggested to be closely correlated with higher cortical neurite density in individuals with ASD, and it is considered that is an excess of synaptic connections in their brain during development compared to that in the mature brain. A previous report demonstrated that ASD is associated with a rapid rate of brain growth from 2 to 4 years of age, after which volume differences gradually decrease with age, resulting in nearly the same brain volume in patients with ASD and TD individuals in adulthood (75). Synaptic pruning is a brain process involving the reduction of excess synapses to allow the establishment of appropriate neural networks during neural development (76–78). Inappropriate synaptic pruning results in an excess of dendritic spines and immature synapses, and consequently in abnormal neural circuits (76). In individuals with ASD, one possibility is that exposure to ACEs has a worse influence on proper synaptic pruning compared to that in TD individuals, which results in excess and abnormal neural circuits. Consequently, ACE-related immature neural function could be a critical factor in the manifestation of PTSD symptoms in individuals with ASD.

This study has several limitations. Even with careful individual explanations by the evaluators, the potential difficulties with the psychological tests may have introduced a bias, especially for participants with ASD. As the CATS uses a self-evaluation questionnaire for rating exposure to ACEs, the possibility of a recall bias among the participants cannot be ruled out, and could have influenced the study findings. Nevertheless, the CATS was used to evaluate ACE severity as it has been well-validated in a previous study (79). Then, in the voxel-based analysis, there were no significant brain regions associated with IES-R-J intrusion subscale scores after controlling for age as a covariate among individuals with ASD. It could be because of the relatively small sample size of the current ASD participants. Additionally, although age of the ASD samples mostly ranged from 20 to 40 years, the effect of aging can influence on the NODDI parameters (80). Then, although we identified certain brain regions associated with each PTSD symptom by voxel-based whole brain analysis in individuals with ASD, we could not evaluate functioning of brain network related to these brain regions. In addition, medication history may have influenced the severity of certain PTSD symptoms in individuals with ASD. Accordingly, future studies to address these limitations are warranted.

In conclusion, our results indicate that ACE-associated higher cortical neurite density is involved in PTSD symptoms in individuals with ASD. Neurodevelopment during childhood and adolescence may be susceptible to ACEs for which severity was closely associated with higher neurite density in individuals with ASD. These findings suggest that functional modification of PTSD-associated brain regions may improve PTSD symptoms in ASD. Moreover, clinical intervention against ACEs may allow for stable neural development, preventing the emergence of PTSD symptoms in ASD.

## Data availability statement

The raw data supporting the conclusions of this article will be made available by the authors, without undue reservation.

## Ethics statement

The studies involving humans were approved by the institutional review board of Nara Medical University. The studies were conducted in accordance with the local legislation and institutional requirements. Written informed consent for participation in this study was provided by the participants’ legal guardians/next of kin.

## Author contributions

SK analyzed and interpreted the imaging data. KM, MT, HY, AM, and HO contributed to the interpretation of the results. TM, YT, TO, and TT contributed to the acquisition of MRI data. RI performed the psychological evaluations. MM contributed to the study conception, design, and interpretation of the results. All authors contributed to the article and approved the submitted version.

## Funding

This work was supported by the Japanese Society for the Promotion of Science KAKENHI (grant numbers 19 K17116 to SK; 16H06403, 16H06400, 16H02666, and 16H05377 to MM), AMED-PRIME (grant number 21gm6310015h0002 to MM), AMED-CREST (grant number 22gm1510009h0001 to MM), AMED (grant number 21wm04250XXs0101 to MM), AMED (grant number 21uk1024002h0002 to MM).

## Conflict of interest

The authors declare that the research was conducted in the absence of any commercial or financial relationships that could be construed as a potential conflict of interest.

## Publisher’s note

All claims expressed in this article are solely those of the authors and do not necessarily represent those of their affiliated organizations, or those of the publisher, the editors and the reviewers. Any product that may be evaluated in this article, or claim that may be made by its manufacturer, is not guaranteed or endorsed by the publisher.

## Footnotes

^1^ http://www.fmrib.ox.ac.uk/fsl/

^2^ https://www.nitrc.org/projects/noddi_toolbox/
