# Supplementary figures and images for: Association of adverse childhood experiences and cortical neurite density alterations with posttraumatic stress disorder symptoms in autism spectrum disorder
Source: Front Psychiatry. 2023 Sep 8;14:1215429. doi: 10.3389/fpsyt.2023.1215429 (PMC10515392; doi:10.3389/fpsyt.2023.1215429)

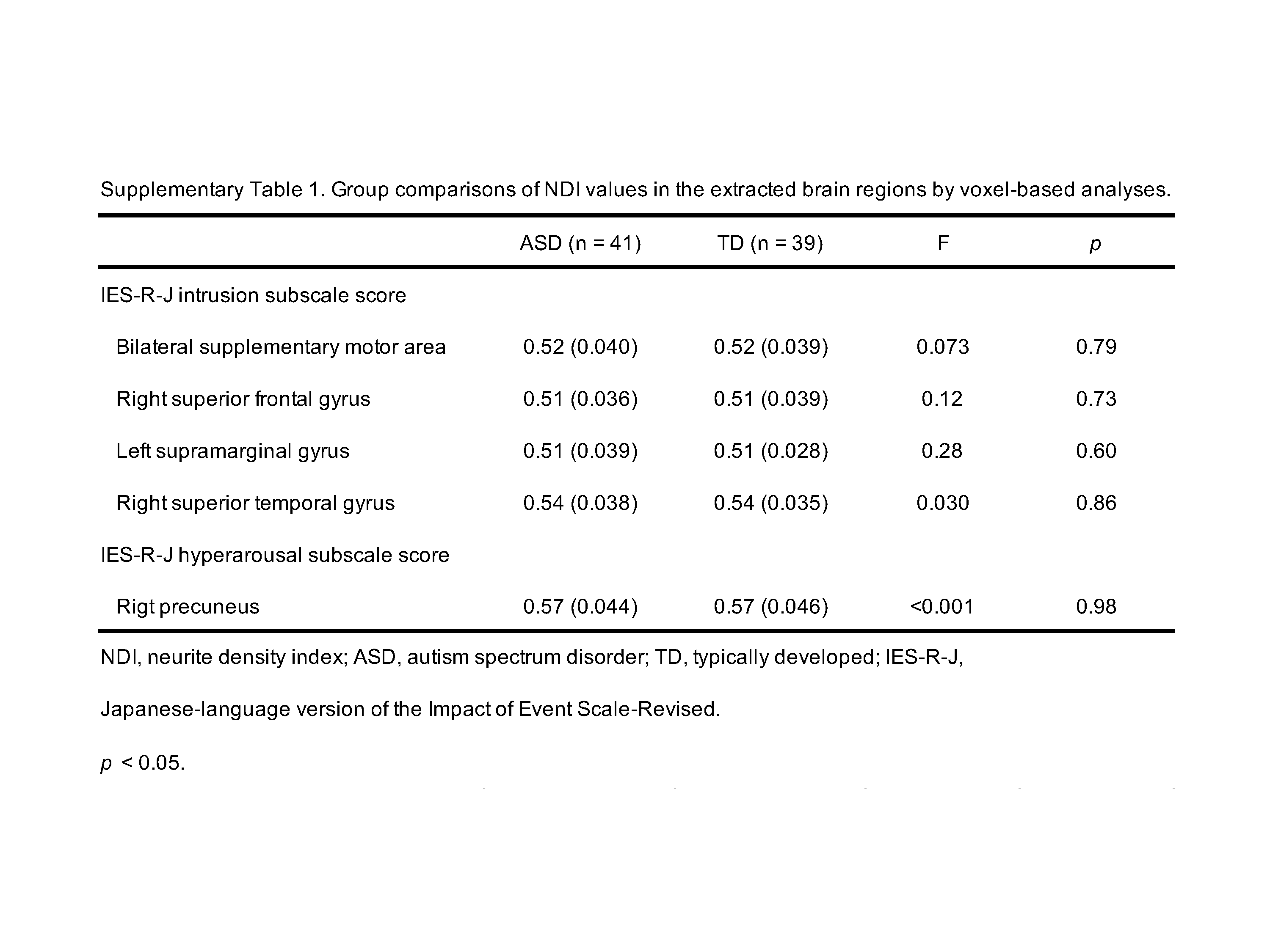

Supplement: Supplementary file 1 [file Image_1.tiff]

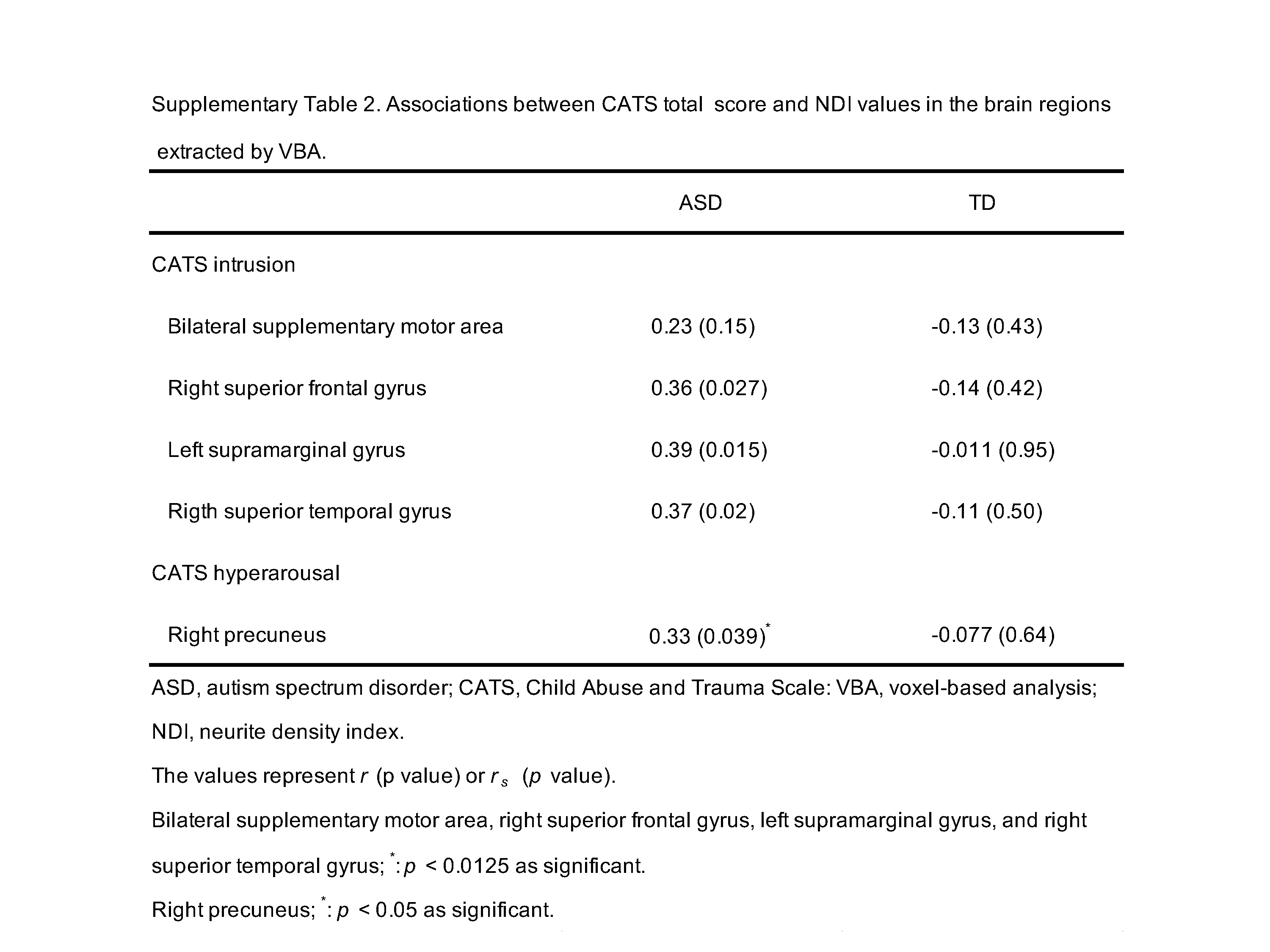

Supplement: Supplementary file 2 [file Image_2.TIFF]

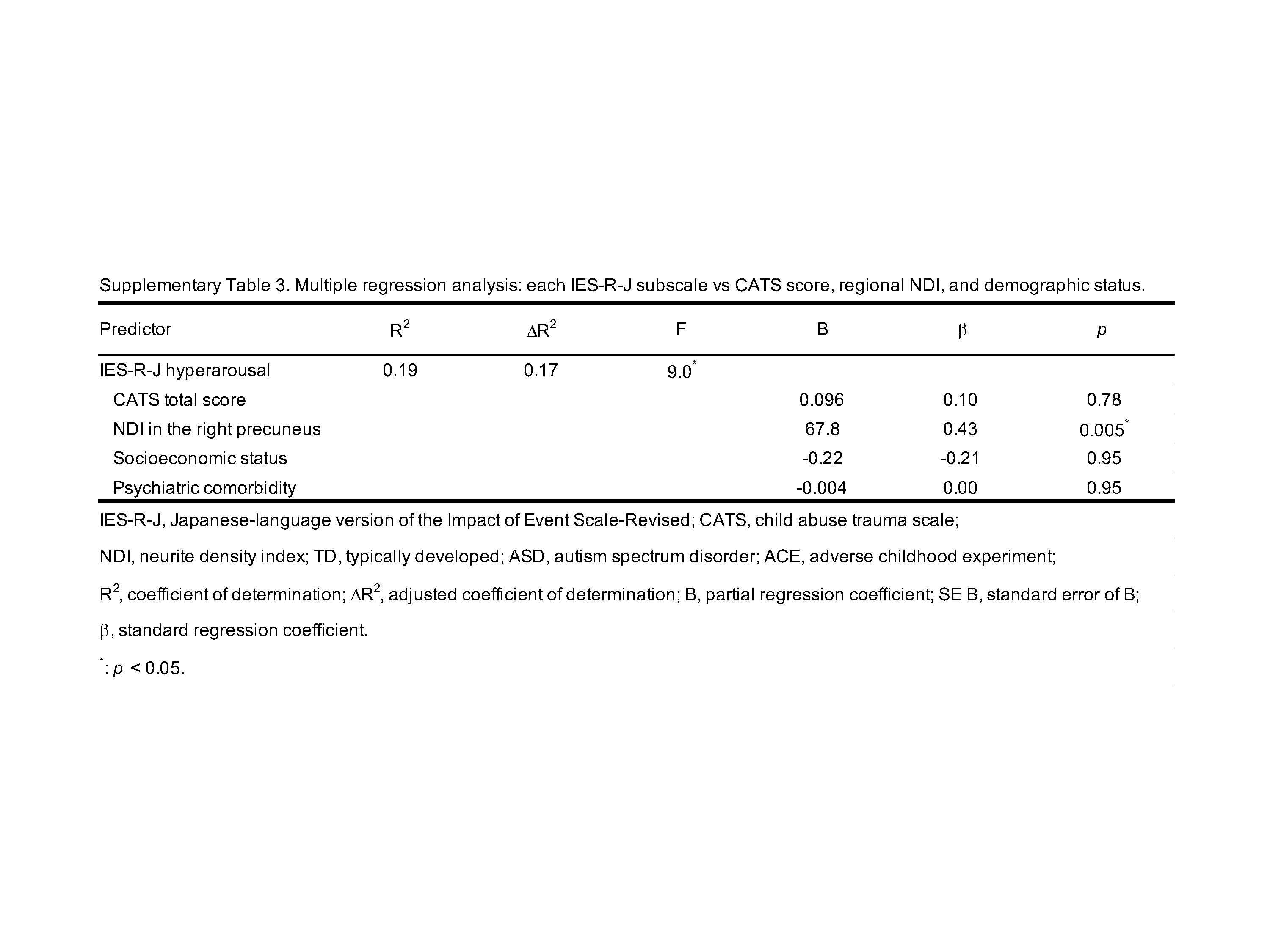

Supplement: Supplementary file 3 [file Image_3.tiff]
